# A randomized, double-blind, placebo-controlled trial for Yi-Qi Hua-Yu tong-sui granule in the treatment of mild or moderate cervical spondylotic myelopathy

**DOI:** 10.1097/MD.0000000000021776

**Published:** 2020-08-14

**Authors:** Chongqing Xu, Xiaoning Zhou, Zhengyi Tong, Junming Ma, Jie Ye, Jinhai Xu, Wen Mo

**Affiliations:** Department of Orthopaedics, LongHua Hospital, Shanghai University of Traditional Chinese Medicine, Shanghai, China.

**Keywords:** mild or moderate cervical spondylotic myelopathy, randomized controlled trial, traditional Chinese medicine, treatment, Yiqi-Huayu-Tongsui granule

## Abstract

**Background:**

Neck pain, sensory disturbance and motor dysfunction in most patients suffered cervical spondylotic myelopathy (CSM). However, some conservative treatments are limited by their modest effectiveness. In the other hand, surgical treatment is necessary when symptoms are refractory to conservative treatments and neurological function of the patients has deteriorated. Many patients use complementary and alternative medicine, including traditional Chinese medicine, to address their symptoms. The purpose of the present study is to examine effectiveness and safety of Yiqi-Huayu-Tongsui (YQHYTS) granule, a compound traditional Chinese herbal medicine, on symptoms in patients with mild or moderate CSM.

**Methods/Design:**

A randomized, double blinded, placebo-controlled clinical trial to evaluate the efficacy and safety of YQHYTS granule is proposed. 72 patients in Longhua Hospital with the diagnosis of mild or moderate CSM will be randomly allocated into 2 groups, and treated with YQHYTS granule or placebo. The prescription of the trial drugs (YQHYTS granule/placebo) is 20 grams twice a day for 3 months. The primary outcome measurements include visual analog scale, Japanese Orthopedic Association, and Neck Disability Index score. The secondary outcome measurements are electromyogram and Pfirrmann classification.

**Discussion:**

YQHYTS granule has been established and applied in Longhua Hospital for many years. As it has a potential benefit in treating mild or moderate CSM, we designed a double-blind, prospective, randomized controlled trial and would like to publish the results and conclusions later. If YQHYTS granule can alleviate neck pain, sensory disturbance, and even motor dysfunction without adverse effects, it may be a unique strategy for the treatment of mild or moderate CSM.

**Trial registration:**

Chinese Clinical Trial Registry ID: ChiCTR1900028192. Registered 15 December 2019, Available at: http://www.chictr.org.cn/edit.aspx?pid=46913&htm=4

## 1 Introduction

Cervical spondylotic myelopathy (CSM) is a kind of spinal cord dysfunction disease, which is caused by spinal cord compression and / or spinal cord blood supply disorder due to the influence of cervical degenerative changes. This type of cervical spondylosis has a high incidence, a wide range of people, serious symptoms, high disability rate, high operative difficulty and risk.^[1,2]^ Patients with severe or rapid progression of CSM should be treated with early surgery, but most of the patients with early or less severe CSM can be treated with non-surgical treatment.^[3,4]^ Therefore, it is a great challenge for the medical community to seek effective treatment and rehabilitation measures for mild or moderate CSM.

In non-surgical treatment of CSM, the characteristics and advantages of traditional Chinese medicine (TCM) are obvious. As the main TCM treatment of mild or moderate CSM, oral Chinese medicine has definite curative effect.^[5]^

Through long-term clinical observation, Shi Qi, a lifelong professor of Shanghai University of TCM and national famous TCM orthopedic master, found that CSM is similar to Bi-syndrome (a syndrome of TCM) in pathogenesis and syndrome characteristics, that is to say, it's a kind of disease syndrome with main performance of pain, sensory disturbance and even motor dysfunction, which is caused by occlusion of meridians, poor circulation of Qi and blood. The experimental study^[6]^ confirmed that the TCM of YiQi-HuaYu-BuShen can effectively reduce the local inflammatory response of the spinal cord after chronic spinal cord injury and compression, inhibit the apoptosis of neurons and glial cells, and promote the expression of NGF mRNA and BDNF mRNA, suggesting that the TCM of YQHYBS had therapeutic potential against CSM. Therefore, Yiqi-Huayu-Tongsui (YQHYTS) granule was established by our research team to treat mild or moderate CSM, using Radix Astragali Preparata as the principal element, while Codonopsis pilosula, Angelica sinensis, Radix Paeoniae Alba, Rhizoma Chuanxiong, earthworm, Radix Achyranthis Bidentatae and Radix Glycyrrhizae Preparata as adjuvant components.

Since YQHYTS was created, it had definite curative effect and few side effect in clinical use. However, no study indicated the efficacy of YQHYTS in the treatment of mild or moderate CSM as well as the side effect. In regard of this, our study aim to conduct a double blinded, well-designed randomized and placebo-controlled trial to estimate the efficacy and safety of YQHYTS for mild or moderate CSM. The results of this trial will provide us with evidence to support the hypothesis whether the YQHYTS can relieve sensory disturbance, improve motor function and delay or prevent surgery. Considering the inconvenient for the use of decoction and better quality control for drug, we apply YQHYTS granule instead of the decoction in our trial. YQHYTS granule is the raw extraction of YQHYTS decoction, which has the advantage of long-term storage, and the convenience of manufacturing and quality control, and has been widely accepted in hospital for many years.

## 2 Materials and methods

### 2.1 Study design

This study is a randomized, double blinded, placebo-controlled clinical trial. The aim of our study is to evaluate whether YQHYTS combined with MeCobalamin tablets (BoKeBao) is effective in treating patients with mild or moderate CSM. BoKeBao is the basic medicine in this trial. 200 participants with mild or moderate CSM will be recruited from Longhua Hospital affiliated to Shanghai university of TCM. All the patients will be randomized into YQHYTS group or placebo group.

### 2.2 Preparation of YQHYTS granule

The preparation of YQHYTS granule will be manufactured, packaged and labeled by a factory in Shanghai based on good manufacturing practice standard. The crude herbs, including Radix Astragali Preparata (Zhi Huang Qi) 14.5 kg, Codonopsis pilosula (Dang Shen) 21.8 kg, Angelica sinensis (Dang Gui) 13.2 kg, Radix Paeoniae Alba (Bai Shao) 8.7 kg, Rhizoma Chuanxiong (Chuan Xiong) 17.4 kg, earthworm (Di Long) 8.7 kg, Radix Achyranthis Bidentatae (Niu Xi) 8.7 kg and Radix Glycyrrhizae Preparata (Zhi Gan Cao) 8.7 kg were supplied in 1 batch from Longhua Hospital and were kept and stored in a specialized cool and dry place. YQHYTS granule was made as follows:

(1) Extraction, put all herbs listed above in a ceramic container, add 1000 liters distilled water into the container to macerate the herbs for 1 hour, and then boil the mixture at 100°C for 1 hour for the first extraction, and repeat twice to get 3 extractions in total. After that, pour-out the liquid extract, and add 1000 liters distilled water into the container and boiling at 100°C for 1 hour, and the third extraction involved 500 liters distilled water for half an hour boiling time.
(2) Concentration: Mix 3 above extractions together, and concentrate the mixture at 60°C (660 mm Hg) and spray dried to produce a extract powder which are ready to be smashed and screened through mesh size of 80.
(3) At last, the granule were packed at 20 g per bag and stored in a clean room at about 20°C and 50% humidity. YQHYTS granule placebo contains 10% YQHYTS granule and 90% bitterant, lactose edible essence, pigment (such as lemon yellow, caramel pigment and sunset yellow) and starch, which exhibit similar shape, smell, color and taste to YQHYTS granule.

### 2.3 Ethical issues

The trial will be conducted in accordance with the Declaration of Helsinki and Ethical Guidelines for Clinical Research. The trial protocol has been approved by the Research Ethical Committee of Longhua Hosptial, affiliated to Shanghai University of TCM, Shanghai, China (approval Number: 2019LCSY081). And the protocol has been registered on Clinical Trials and Chinese Clinical Trial Registry (ID: ChiCTR1900028192).

### 2.4 Study participants

We will recruit participants from Longhua Hospital through publishing recruitment poster and website advertisements. Informed consent will be obtained from all participants before randomization. The planned recruitment period is 12 months.

### 2.5 Eligibility criteria

#### 2.5.1 Inclusion criteria

Participants who fulfill the list below, are eligible.

(1) Signed informed consent form.
(2) The patients who meet the diagnostic criteria belong to mild or moderate CSM.^[7]^
(3) Male or female aged 40 to 75 years.
(4) The course of disease is more than 3 months.
(5) Volunteer to receive the treatment (YQHYTS granule or placebo drugs combined with Bokebao).
(6) The patients didn’t received glucocorticoids orally, intravenously, intramuscularly or in soft tissue 4 weeks before accepting the test drug.

#### 2.5.2 Exclusion criteria

Participants who meet the criteria as follows should be excluded.

(1) Those who have participated in or are participating in other clinical researches in the past 3 months.
(2) The patients who had been treated with regular non-surgical treatment for at least 6 months to no avail and had more severe symptoms.
(3) The muscle strength decreased significantly in a short period of time, and the muscle strength was lower than grade III.
(4) Patients with history of severe cervical trauma and cervical surgery.
(5) Pregnant women, lactating women and psychopaths.
(6) Patients with heart, liver, kidney or hematopoiesis impairment.
(7) People who are allergic to various drugs.
(8) Mentally or legally disabled.
(9) Those who have a history of opioid analgesics, sedative hypnotics and alcohol abuse.
(10) Patients with serious primary diseases such as liver, kidney, hematopoietic system, endocrine system, cardio cerebrovascular system, nervous system, tuberculosis, vertebral deformity, malignant tumor and psychosis.
(11) Those who do not read or write in Chinese.

### 2.6 Sample size calculation

We calculated the sample size on the basis of our preliminary study, the comparison treatment (YQHYTS granule) versus physical therapy, from December 2018 to May 2019, with equal allocation in 2 groups and 4 repeated measurements. As dysfunction is the main complaint in CMS, we chose Japanese Orthopedic Association (JOA) score for calculating the sample size. The result indicated that YQHYTS group could achieve 52.8% JOA improvement while the physical therapy group only achieve 25.7%. According to the formula of the rate in completely random design, 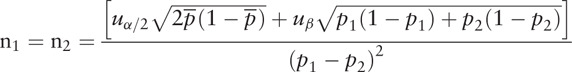


among which, n_1_ and n_2_ are the number of YQHYTS and the placebo group, *u*_*ɑ*/2_ = 1.96 when type 1 error is 0.05, *u*_*β*_ = 1.282 when type II error is 0.1 in 2-sided tests. 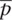 is the mean of *p*_1_ and *p*_2_,^[8]^ it is estimated that approximately 30 participants per group was needed to achieve 90% power and a (2-sided) 5% significance level in detecting treatment differences. Therefore, the sample size was eventually identified as a total of 72 patients (36 per group), considering 20% loss to follow up.

### 2.7 Interventions

Patients in YQHYTS group will receive both YQHYTS granule (20 g, twice a day) and BoKeBao (5 mg per day) for 3 months, while patients in the other group will receive YQHYTS granule placebo (20 g, twice a day) and the same dose of BoKeBao as the YQHYTS group. Both YQHYTS granule and placebo should be dissolved in 200 milliliters hot water as the instruction and then the solution can be taken by patients orally. While patients in the placebo group will take YQHYTS placebo as the same way as the YQHYTS group. In addition, no other drug is allowed.

The duration of the intervention is 3 months, and that of the follow-up is 9 months. Study visits will take place at baseline, at week 2 and 4, and at month 3, 6 and 12. Each patient will be asked to visit at the given time point mentioned above in 3 days.

### 2.8 Randomization and allocation

Block randomization will be carried out in a 1:1 ratio through Statistical Product and Service Solutions 22.0 to either the YQHYTS group or the placebo group randomly. Randomization will be generated by the credible pharmaceutical factory, which provides us both YQHYTS granule and the placebo. When a participant is recruited in, the investigator will provide the pharmaceutical factory a number (made according to participating order), the pharmaceutical factory will randomly post the YQHYTS granule (or the placebo) to the participant according to the random number list. The provided number and the corresponding number list will be recorded and will be kept in a locked cabinet in an office of the pharmaceutical factory.

### 2.9 Blinding

We will use the third-party evaluation method. Special personnel shall be responsible for the test index detection, data collection and statistics. Neither the tester nor the statistician knows the test grouping and specific operation. Two persons not involved in this project will be assigned, 1 will record all the observation and evaluation indexes, and the other will be responsible for the quality monitoring of the whole process of the project, in addition, the data collection, storage and analysis will be in the charge of the trained special person, and the therapists will not be involved in the work. The staff in the pharmaceutical factory will not participant in the trials, and the investigator, doctors, nurses, outcome measuring person, statisticians and the participants will have no idea about the group information. Group assignment will not be revealed until the whole trial is finished, including all the statistics work.

### 2.10 Outcome Measurements

#### 2.10.1 Primary study outcomes

Will be recorded at baseline, the 2nd week, the 4th week, the 3rd month, the 6th month and the 12th month of follow-up.

(1) visual analog scale (VAS) score: VAS is the most common method to evaluate pain, ranges from 0 mm (no pain) to 100 mm (worst pain ever experienced), which is usually a horizontal line, set by word descriptors at each end. Patients chose 1 point which can represents their current pain degree on VAS line.^[9]^
(2) JOA score: JOA scores include upper limb motor function, lower limb motor function, sensation, bladder function. The JOA score ranges from 0 to 17. Lower score indicates more obvious dysfunction.
(3) Neck Disability Index score: Neck Disability Index is a composite of functional status including 2 parts, 10 items in total: neck pain and related symptoms, and the ability of daily activities. This will be filled in by the participants according to their own situation. The score of each item ranges from 0 to 5. Higher score indicates severer dysfunction.

#### 2.10.2 Secondary study outcomes

Will be recorded at baseline and the 6th month of follow-up.

(1) Electromyogram: It is the bioelectric pattern of muscle recorded by electromyograph. It is of great significance to evaluate human activities in man-machine system. They are closely related to muscle load intensity. According to the EMG description and the diagnosis report, we can determine the progress or improvement of the patient's condition.
(2) Pfirrmann classification: This classification is established by Pfirrmann. It identifies and grades the degree of lumbar disc degeneration according to patient's T2-weight imaging of MRI. There are 8 grades in total.

### 2.11 Adverse events

In the period of this trial, all the reported adverse events from participants will be record no matter related to YQHYTS granule or not. The relationship of adverse events and YQHYTS granule will be studied after adequate treatment measures, and all reported adverse events will be analyzed in spite of the researchers’ assessments of causality, and will be categorized according to the Medical Dictionary for Regulatory Activities (MedDRA, Version 8.1J).

### 2.12 Safety assessments

In order to assess the safety of YQHYTS granule, the following tests will be performed and monitored on all participants at the baseline (week 0) and after treatment (month 3): the blood routine, urine routine, feces routine, kidney and liver function of patients. At the same time, the treating clinicians will record all adverse effects including serious adverse effects. If there is any adverse event, clinicians will adjust treatment strategy immediately in accordance with the protocol and will provide emergency services in case of serious events. All adverse events will be reported to the Institutional Review Board within 24 hours. Blinding will be broken to offer the whole treating history for adopting the most adequate procedures.

### 2.13 Study schedule

**Figure 1:**
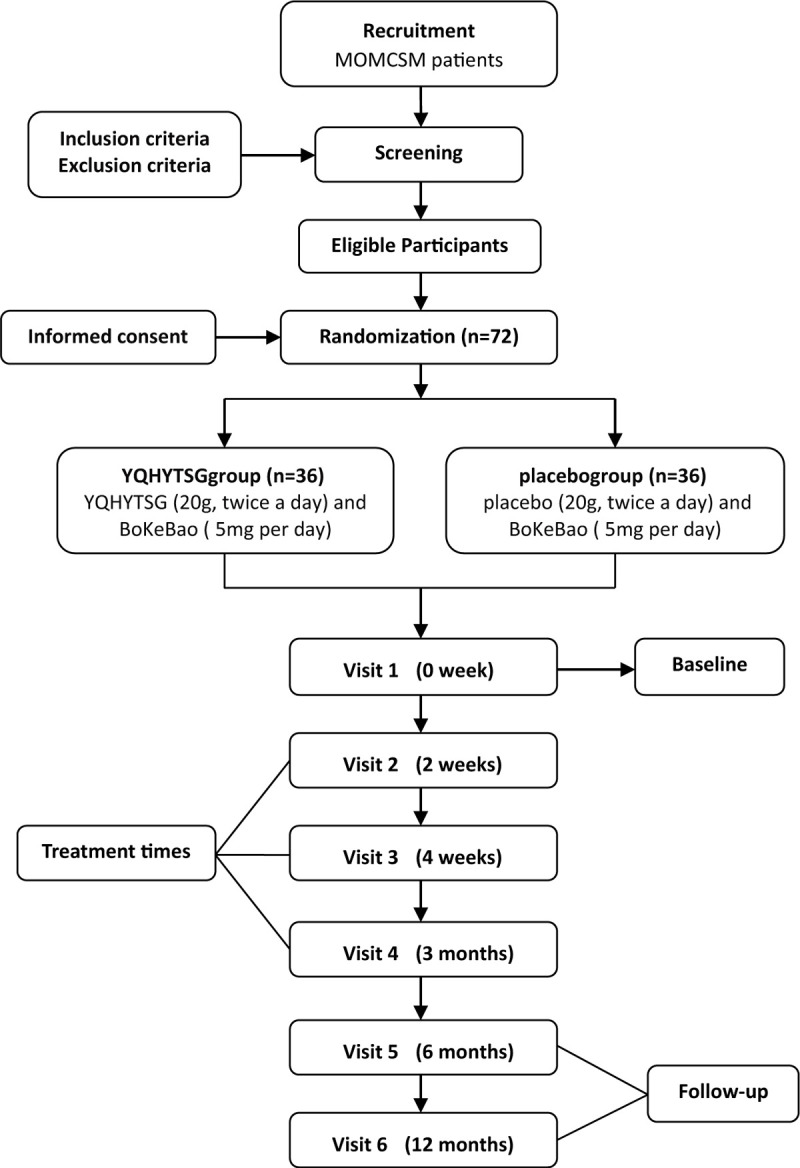
Project overview. MOMCSM = mild or moderate CSM, YQHYTSG = Yiqi-Huayu-Tongsui granule.

Recruitment will start in June 2020, and is expected to end in June 2021, and all the follow-up of participants will be finished before 30th, June 2022. The schedule of enrollment and evaluation is shown in Table 1, and the flow chart of participant in this trial is shown in Figure 1.

**Table 1:**
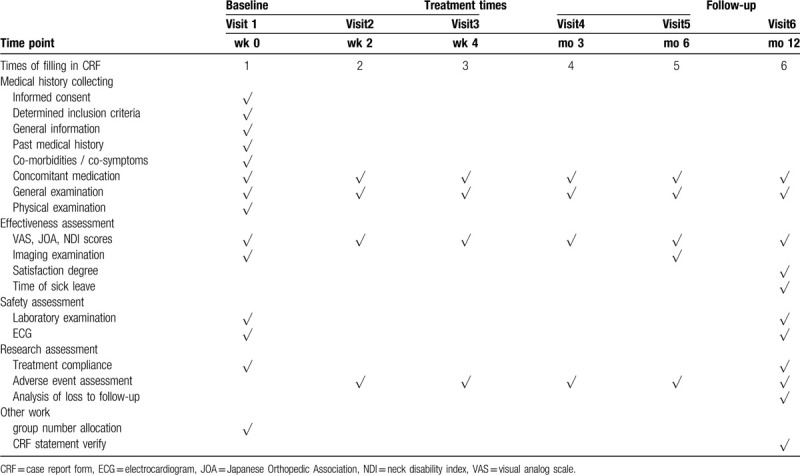
Study schedule of clinical trial (6 mo).

### 2.14 Data collection and monitoring

This is a 12-months clinical trial, in which participants need to take research medication for 3 months with 9 months’ follow-up. They will receive 6 repeated assessment (the baseline, week 2, week 4, month 3, month 6 and month 12). Longhua Hospital affiliated to Shanghai university of TCM is responsible for monitoring and quality control.

### 2.15 Statistical Analysis

Efficacy and safety analyses will be conducted according to the intention-to-treat principle. Last-observation-carried-forward method will be applied in the missing values. All statistical analyses will be performed using Statistical Product and Service Solutions (version 22.0). *P*-value < .05 will be defined as statistical significance. Means and standard deviations will be described in the continuous variable such as demographic, and outcome variables, while the percentages will be used for categorical variables such as the rate. Continuous variable followed the normal distribution will be calculated by Student *t*-tests, otherwise non-parametric test will be used to compare group differences. We will compare the baseline characteristics of the 2 groups, and if there is no comparability, we will conduct a secondary analysis to adjust the difference. The primary outcome, the amount of pain, was compared between the 2 groups using survival analysis and repeated measures of variance analysis.

## 3 Discussion

With the growth of age, affected by genetic and environmental factors, the degenerative changes of intervertebral disc and small joint of cervical vertebrae and formation of osteophyte at the edge of vertebral body may cause spinal stenosis, chronic compression of the spinal cord, resulting in neck pain, limb dysfunction and even paralysis. The latest research shows that the incidence rate of CSM is about 1.6/10000,^[10]^ and the highest proportion is in Asian men and European white women.^[11]^ The main clinical treatment of CSM includes conservative treatment and surgical decompression. A recent study shows that non-operative treatment is not suitable for patients with moderate and severe CSM, for there is not enough evidence to show that non-operative treatment can change the natural history of CSM. It is necessary to closely observe whether the neurological function of the patients has deteriorated if conservative treatment is used for patients with mild CSM.^[12]^ The non-surgical treatment mainly includes cervical spine immobilization, anti-inflammatory and analgesic drugs, neurotrophic medicine, physical therapy, occupational therapy and other rehabilitation therapies. Analgesics, such as Aspirin and Celecoxib, appear to be relatively safe, but gastrointestinal reactions are reported to occur frequently.^[13]^ Mecobalamin, a kind of neurotrophic medicine, can relieve neck pain by stimulating the inhibition of neurodegenerative changes and improving excitatory nerve fibers.[Okada K, Tanaka H, Temporin K, et al Methylcobalamin increases Erk1/2 and Akt activities through the methylation cycle and promotes nerve regeneration in a rat sciatic nerve injury model. Exp Neurol. 2010; 222:191–203.] In recent years, modern medical and TCM clinical researchers have carried out systematic study in the prevention, treatment, diagnosis and other aspects of CSM, including randomized controlled trial, systematic review and the law of syndrome differentiation.

Nowadays, TCM has increasingly attracted world's attention. Many people gradually recognize the effectiveness of Chinese herb. The study by Mo W et al^[14]^ indicated that Yiqi-Huayu-Bushen decoction could promote the recovery of spinal cord function, improve the intracellular environment and delay the degeneration of disc, and concluded that the relevant TCM had definite therapeutic effect on CSM, meanwhile longer treatment brought more significant effect. As a similar kind of TCM decoction, YQHYTS has been used in our hospital for many years with clear therapeutic effect on mild or moderate CSM.

Though there are only 8 herbals in this prescription, the compatibility of them is precise and appropriate. Based on the theory of TCM, the components of a prescription have the functions of leading, coordinating, assisting and guiding. Radix Astragali Preparata and Codonopsis pilosula play a leading role in YQHYTS granule, Angelica sinensis, Radix Paeoniae Alba and Rhizoma Chuanxiong play a coordinating role, earthworm and Radix Achyranthis Bidentatae play an assisting role, while Radix Glycyrrhizae Preparata plays a guiding role. All components of Qishu pill are considered to be beneficial to patients with pain, sensory disturbance and motor dysfunction. The ability of AS-IV in Radix Astragali Preparata to inhibit NF-qB pathway may be 1 of the potential mechanisms of its anti-inflammatory effect in vivo.^[15]^ Codonopsis pilosula polysaccharides can clear away free radicals, protect vascular endothelial cells and weaken the stress-induced senescenceIt caused by oxidation, which may reduce the injury of spinal cord blood vessels.^[16]^ The study by Li RC^[17]^ showed that Angelica injection may have partial analgesic effect by inhibiting the activation of spinal astrocytes. Total glucosides of paeony in Radix Paeoniae Alba can alleviate inflammatory demyelination in mice, reduce infiltration of inflammatory cells, decrease vacuolation of spinal cord, and axonal injury.^[18]^ Rhizoma Chuanxiong interferes with ROS production and Ca2^+^ influx through G protein regulation, in order to prevent the up-regulation of neutrophil activated Mac-1, and plays an anti-inflammatory role.^[19]^ The aqueous extract of earthworm can significantly prolong the formation time of fibrin thrombus and platelet thrombus in vivo, thus improve blood flow.^[20]^ Achyranthes bidentata polypeptide has neuroprotective effect, it can reduce the apoptosis of spinal cord and dorsal root ganglion cells induced by unilateral sciatic nerve transection through inhibiting the expression of apoptotic gene Bax and promoting the expression of antiapoptotic gene bcl-2.^[21]^ Radix Glycyrrhizae Preparata has been demonstrated to have strong anti-inflammatory effects.^[22]^ Anti-inflammatory effects of Radix Glycyrrhizae Preparata extracts on TPA-induced acute inflammation in mice have been reported.^[23]^

While the public praise is affirmative, we still need solid evidence to show the effectiveness and safety. We had searched a list of databases including PubMed, the Cochrane Library, EMBASE, Sinomed, CNKI, Wan Fang Data, and Clinical Trials by the end of 2019 before we designed this trial. There's no clear evidence about the effectiveness and safety of YQHYTS granule on the treatment of mild or moderate CSM. Accordingly, we resolved to design a randomized, controlled clinical trial for the further research of the effectiveness and safety. Since Mecobalamin is a first-line medication for CSM, and the evidence about the effectiveness of YQHYTS granule is insufficient, we combine YQHYTS granule with Mecobalamin to keep patient compliance and ethical permission.

As far as we know, this is the first well designed RCT about the combined of YQHYTS granule with Mecobalamin on the treatment of mild or moderate CSM. This study is to prove whether YQHYTS granule combined with Mecobalamin in treating mild or moderate CSM is better than the single use of Mecobalamin. If the study proves the efficacy of the experimental group is significantly better than the control group, we can provide a better guide and therapeutic regimen for the treatment of mild or moderate CSM, furthermore, we can also observe the repair effect of spinal cord and the long-term curative effect, and explore its mechanism.

## Author contributions

Chongqing Xu is the first author of this manuscript, contributing to the design, conduct of the trials, and drafting of the manuscript. All authors participated in the design of the study and performed the trial. Jinhai Xu and Wen Mo are the co-corresponding authors of this manuscript, contributing equally to the supervision and coordination of the clinical trial. All authors read and approved the final manuscript.

**Investigation**: Chongqing Xu, Xiaoning Zhou.

**Methodology**: Jinhai Xu, Zhengyi Tong.

**Project administration**: Jinhai Xu.

**Supervision**: Wen Mo, Junming Ma, Jie Ye.

**Writing** – **original draft**: Chongqing Xu.

**Writing – review & editing**: Chongqing Xu, Jinhai Xu.
